# LncRNA AFAP-AS1 promotes anaplastic thyroid cancer progression by sponging miR-155-5p through ETS1/ERK pathway

**DOI:** 10.1080/21655979.2021.1918537

**Published:** 2021-05-17

**Authors:** MingLiang Ning, Shaojie Qin, Jia Tian, Yuchen Wang, Qingyuan Liu

**Affiliations:** a The Third Department of Surgical Oncology, General Hospital of Ningxia Medical University, Yinchuan, China; b Key Laboratory of Fertility Preservation and Maintenance of Ministry of Education, General Hospital of Ningxia Medical University, Yinchuan, China

**Keywords:** LncRNA AFAP-AS1, proliferation, miR-155-5p, ETS1, anaplastic thyroid cancer

## Abstract

Anaplastic thyroid cancer (ATC) is the most common malignant endocrine tumors which resist to majority treatment. Thus, there is impelling need to figure out the mechanism of progress of ATC. In this study, we explored the function and mechanism of lncRNA actin filamentin-1 antisense RNA (AFAP-AS1) which provided a new biomarker for ATC. Viabilities and apoptosis were tested by CCK-8, colony formation and flow cytometry. The interactions between miR-155-5p and AFAP-AS1 or ETS1 was detected by luciferase reporter assays. ETS proto-oncogene1/mitogen-activated protein kinase1 (ETS1/ERK) pathway was assessed by Western blot. Xenograft models were built to confirm the function of AFAP-AS1 *in vivo*. Firstly, we showed that relative RNA expression of AFAP-AS1 in ATC cells was higher than in immortalized thyroid cells. Next, AFAP-AS1 was verified as an oncogene in ATC since knock-down of AFAP-AS1 inhibited cell proliferation and accelerated apoptosis. In addition, miR-155-5p was negatively regulated by AFAP-AS1. Moreover, AFAP-AS1 regulated ETS1/ERK pathway by sponging miR-155-5p. Finally, we confirmed knock-down of AFAP-AS1 significantly suppressed tumor proliferation *in vivo*. Our research proved that AFAP-AS1 could facilitate progression of thyroid cancer sponging miR-155-5p through ETS1/ERK pathway.

## Graphical abstract

**Figure:**
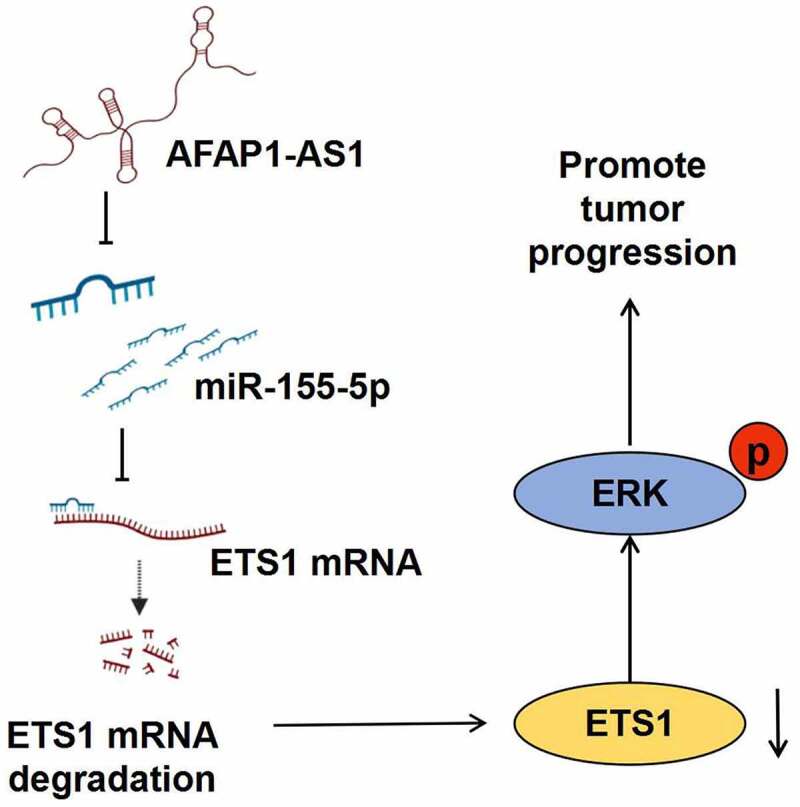

## 1. Introduction

Thyroid cancer, the most common endocrine malignant tumor, became more prevalently in recent years, especially among women [1,2]. Pathologically, most thyroid cancer deriving from follicular epithelium cell divided into papillary thyroid cancer, follicular thyroid cancer and anaplastic thyroid cancer. Although patients suffering from papillary thyroid cancer or follicular thyroid cancer develop good prognosis, the mortality rate for those who progress to anaplastic thyroid cancer is high [3,4]. Ultrasound and then fine needle biopsy are used to detect ATC. The traditional therapy that contains thyroidectomy with hormone and radioiodine treatments for ATC does not improve the fatal outcome of patients [5]. Although there was study showing that ATC patients who received chemotherapy combined with radiotherapy improve the survival duration compared to radio-treatment alone. These patients still develop to cachexia eventually [6]. Moreover, some second-line treatments target to specific gene which induce progression of ATC are also used, for instance, tyrosine kinase inhibitors, anti-angiogenic drugs and multi-kinase inhibitors [7]. However, these molecular targeted treatments do not work well due to drug resistance. Thus, there is impelling need to figure out the mechanism of progress of ATC [8].

Long non-coding RNAs (lncRNAs) is a class of transcripts which present insufficient protein coding capacity ranging from 200 to multiple nucleotides in length. It has been demonstrated that lncRNAs played an important role in different biological activity, such as post transcription of gene, cell proliferation and tumor development by adsorbing miRNA as sponges [9,10]. The lncRNA actin filamentin-1 antisense RNA 1 (AFAP-AS1) is located on chromosome 4p16.1 consisting of 6800 nucleotides in length that took part in proliferation and differentiation of various types of tumor, such as gastric cancer, nasopharyngeal carcinoma, non-small cell lung cancer, and etc [11–13]. Meanwhile, AFAP-AS1 can reflect the prognosis as a biomarker in several types of cancer including esophageal carcinoma, breast cancer etc [14]. However, there is limited study exploring the function and mechanism of AFAP-AS1 in thyroid cancer, especially ATC.

MicroRNAs (miRNAs) belonging to a class of small non-coding RNA containing 18–25 nucleotides, which completely or partially bind the sequence of target genes, inhibit protein translation by post-transcription regulation [15,16]. Multiple target genes can be regulated by one single miRNA, while different miRNAs are able to affect the same gene to control protein suppression. It has been reported that various miRNAs participated in the progression of cancer. For example, Sun Y. et al showed that miR-222-3p influenced metastasis of prostate cancer [17]. Meanwhile, there was study demonstrating that miR-574-3p participated in TGF-βpathway and then enhanced the progression of colorectal cancer [18]. Recent reports have been showed that miR-155-5p also played a crucial role in cancer progression [19]. Up to now, it has demonstrated that miR-155-5p influenced tumor proliferation and metastasis competing with AFAP-AS1 to regulate target gene expression [20].

EST1 is a well-known tumorigenic transcription factor which regulates the cytokine and chemokine genes in a wide variety of different physiological and pathological activity directly [21]. It has been reported that ETS1 not only influence mitogen-activated protein kinase1 (ERK) expression but also its phosphorylation [22,23]. ERK, extracellular signal-regulated kinase, affects cell growth by controlling many proteins through its kinase activity or transcription factor function. Current researches showed that the ERK signal pathway played a crucial part in the pathological behavior of anaplastic thyroid cancer [24]. Meanwhile, there were *in vitro* and *in vivo* evidences demonstrating that patients who suffered from anaplastic thyroid cancer responded to therapy differently because of treatment resistance through ERK signal pathway [25]. Thus, the function of ERK directly influence the prognosis of anaplastic thyroid cancer.

Here in this study, we aim to explore the role and function of lncRNA AFAP-AS1 in thyroid cancer. Due to its higher expression in tumor cells than in immortalized cells, we hypothesized that lncRNA AFAP-AS1 might played oncogene role in anaplastic thyroid cancer. Subsequently, a series of bioinformatic technologies and molecular experiments indicated that lncRNA AFAP-AS1 exerted its oncogene function through miR-155-5p/ETS1/Erk axis in anaplastic thyroid cancer.

## 2. Methods and materials

### 2.1. Cells culture

Human immortalized thyroid cell, Nthy-ori-3-1, and human anaplastic thyroid cancer cell lines, C643, 8305 C and 8505 C were purchased from Cell Bank of Type Culture Collection of Chinese Academy of Sciences (Shanghai, China). Cells were cultured in the RPMI 1640 medium with 10% fetal bovine serum, 1 mM pyruvate sodium and 0.5 mM non-essential amino acid (Gibco). All cells were routinely incubated at 37°C in a humidified 5% CO2 condition.

### 2.2. Cell transfections

Lentivirus encoding AFAP-AS1-shRNA and control shRNA were designed from HanBio Biotechnology Co., Ltd. 8305 C and 8505 C cells were transfected at 50% confluence with a final lentivirus multiplicity of infection (MOI) of 10–100 following the instructions of the manufacturer.

After cells were grown up to 60% confluence, cells were transfected with a small interfering RNA targeting ETS1 (si-ETS1; 100 pmol), a small interfering RNA targeting no specific gene (si-ETS1; 100 pmol), a miR-155-5p mimic (100 pmol), a miR-NC (100 pmol), a miR-155-5p inhibitor (100 pmol) and miR-inhibitor-NC (100 pmol) by using Lipofectamine 3000 (Invitrogen, USA). These siRNAs and miRNAs are all from Ribo Biotechnology Co., Ltd.

### 2.3. Cell viabilities

1000–3000 transfected 8305 C cells or 8505 C cells were seeded in 96-well plates. After inoculation every 24 hours, 10 μL Cell Counting Kit-8 (CCK8; Beyotime Institute of Biotechnology, Shanghai, China) solution was pipetted and cultured for another four hours at 37°C 5% CO_2_ incubator. Absorbance was measured at 450 nm through a Microplate Reader (Bio-Rad, USA).

### 2.4. Colony formation assays

1000 cells were seeded into a 6-well plate. After one week, the cells were washed with PBS, then fixed with methanol and stained with 0.1% crystal violet solution. Finally, we counted numbers of colony under an inverted microscope. Colonies were counted and colony surface area was quantified using ImageJ.

### 2.5. Apoptosis assays

Cells were transfected at 50%-60% confluence with sh-AFAP-AS1, miR-155-5p mimic and miR-155-5p inhibitor. Apoptotic cells were analyzed through Annexin V-FITC/PI Kit(Invitrogen, USA) according to the manufaturer’s protocol. Cells were washed, re-suspended, and stained by 5 μL of Annexin VFITC and 10 μL of PI. After incubation in dark environment for 15 minutes, cells were detected through a Flow Cytometer (Beckman Coulter, USA).

### 2.6. qRT-PCR

We extracted total RNA with TRIzol reagent (Invitrogen). Next, cDNAs were produced with the RNA templates by using a reverse transcription kit (Invitrogen). Finally, qRT-PCR analysis was performed on a CFX96 Thermal Cycler Dice^TM^ real-time PCR system with SYBR Premix Ex Taq II (TaKaRa, Dalian, China). GAPDH and U6 were used as reference gene, respectively. The sequences of primers are listed in Table 1. The relative expressions were calculated by using 2^−ΔΔCt^ method.

**Table 1.** The sequences of specific primers

Gene name	Primer sequence (5ʹ to3ʹ)
lncAFAP1-AS1	Forward: 5′-TCGCTCAATGGAGTGACGGCA-3′ Reverse: 5′-CGGCTGAGACCGCTGAGAACT-3′
miR-155-5p	Forward: 5′-GTAACCCGTTGAACCCCATT-3′
	Reverse: 5′-CCATCCAATCGGTAGTAGCG-3′
ETS1	Forward: 5ʹ- GAGTCAACCCAGCCTATCCAGA-3ʹ Reverse: 5ʹ- GAGCGTCTGATAGGACTCTGTG −3’
GAPDH	Forward: 5ʹ-ATCCACGGGAGAGCGACAT-3ʹ Reverse: 5ʹ-CAGCTGCTTGTAAAGTGGAC-3’
U6	Forward: 5ʹ-ACAGATCTGTCGGTGTGGCAC-3ʹ Reverse: 5ʹ-GGCCCCGGATTATCCGACATTC-3’

### 2.7. Fluorescent in situ hybridization (FISH)

Fluorescent in situ hybridization (FISH) assay was carried out using Ribo™ Fluorescent in Situ Hybridization Kit (RiboBio, Guangzhou, China). DAPI stained nucleus, the lncAFAP-AS1 probe was labeled with Cy3 and hybridization was performed according to the manufacturer’s instructions. Briefly, 8305 C and 8505 C cells were cultured in 35 mm confocal dishes at 80% density. The cells were washed with PBS twice, fixed (10 min with 4% formaldehyde), and permeabilized with 70% ethanol at room temperature. Prior to the hybridization, the cells were incubated with prehybridization solution at 37°C for 30 min. Cells were then hybridized with the probes for AFAP-AS1 probe (0.5 ng/µl) in hybridization solution at 4°C overnight. After hybridization, the cells were washed in washing buffer at room temperature for 5 min twice (with the addition of DAPI in the second wash) and then in PBS twice. The confocal laser-scanning microscope (Leica Microsystems, Wetzlar, Germany) was used to detect fluorescence.

### 2.8. Luciferase reporter assays

LncAFAP-AS1 Wild Type (WT) or LncAFAP-AS1 mutant type (MUT) and ETS1 3ʹUTR Wild Type (WT) or ETS1 3ʹUTR mutant type (MUT)were inserted into luciferase report gene vectors (pRL-TK, Promega). The sequences of constructs were confirmed by Sangon Biotech Co., Ltd. Next these plasmids were co-transfected with miR-155-5p mimic or miR-155-5p inhibitor or miR-NC (miR-negative control) or inhibitor-NC (inhibitor-negative control) into 8305 C cells for 48 h. The relative luciferase activity was assessed using Dual-Luciferase Reporter Assay System (Promega). All plasmids were made from Genepharma, China.

### 2.9. Western blotting

Cell and tissue were lysed by RIPA Buffer containing protease inhibitors (Beyotime, Shanghai, China). Equal amounts of protein were separated by 10% sodium dodecyl sulfate polyacrylamide gel electrophoresis and transferred to PVDF membrane (Bio-Rad, USA). The PVDF membrane with protein was blocked into 5% bovine serum albumin (BSA) solution for 2 hour at room temperature. Next we incubated the PVDF membrane with anti-ETS1 (1:1000, abcam, UK), anti-p-ERK (1:1000, abcam, UK), anti-t-ERK (1:1000, abcam, UK) and anti-GAPDH (1:1000, abcam, UK) overnight at 4°C. Next, we incubated the PVDF membrane with goat anti-rabbit IgG secondary antibody for 1 hour at room temperature. Finally, the immunoblotting signals were detected through chemiluminescence system. GAPDH was an internal control.

### 2.10. Animal experiments

A total of 6 × 10^6^ sh-AFAP-AS1 or sh-NC transfected 8305 C cells were injected into six-week-old female nude mice subcutaneously (n = 5 per group). Tumor volume was measured by a vernier caliper every 3 days following the formula: volume = 1/2 × length× width^2^. We conducted animal experiments which followed the Ethics Committee of General Hospital of Ningxia and the guidelines of the National Institutes of Health’s (NIH) ‘Guide for the Care and Use of Laboratory Animals’ (8th edition).

### 2.11. Immunohistochemistry (IHC)

After fixing in 4% formalin for 48 h, the tumor xenografts were then embedded in paraffin and sectioned at 4-μm. Subsequently, the sections were deparaffinized, rehydrated and incubated with anti-Ki67 (1:200, Abcam, Cambridge, UK) and anti-pERK antibody at 4°C overnight. Then, we added biotinylated secondary antibodies to tissues for 1 h at room temperature and then visualized with diaminobenzidine substrate (Sigma-Aldrich, St Louis, MO, USA). Immunohistochemistry (IHC) images were taken using an Olympus microscope.

### 2.12. Statistical analysis

Experiments were performed in triplicates. Data analysis was performed through Graphpad Prism 6.0 (GraphPad, USA). The data were shown as mean ± SD. P < .05 considered as statistically significant.

## 3. Results

In our study, we firstly observed that AFAP-AS1 was over-expressed in anaplastic thyroid cancer cells compared to immortalized thyroid cells. Then to explore the function of AFAP-AS1, we depleted AFAP-AS1. The results showed that depletion of AFAP-AS1 inhibited proliferation and facilitated apoptosis. Next, luciferase report assays demonstrate that AFAP-AS1 influenced tumor progress by negatively regulating targeted miR-155-5p. Finally, we proved that miR-155-5p controlled Erk phosphorylation by binding to classic transcription factor ETS1. Thus, AFAP-AS1/miR-155-5p/ETS1/Erk axis was demonstrated to influence the progression of anaplastic thyroid cancer.

### 3.1. Expression and location of lncRNA AFAP-AS1 in thyroid cancer cells

**Figure 1.**
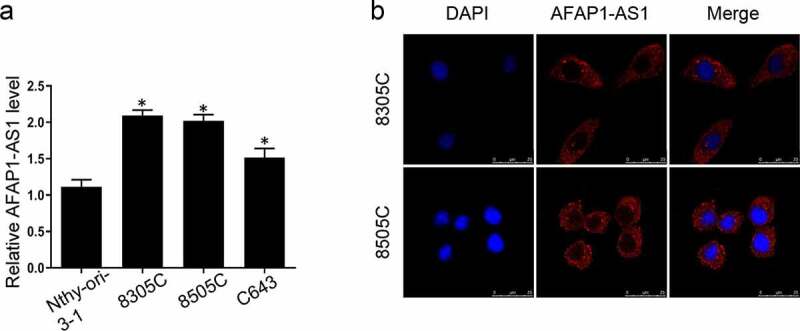
Overexpression of lncRNA AFAP-AS1 in thyroid cancer cells and tissues. (a), RT-qPCR results showed the expression of AFAP-AS1 in anaplastic thyroid cancer cell lines 8305 C, 8505 C, C643 and immortalized and non-tumorigenic thyroid cell Nthy-ori-3-1. (b), FISH experiments were used to detect the location of AFAP-AS1. DAPI was used to stain nuclear. Cy3 fluorescent dye labeled AFAP-AS1. Scale bar = 25 µm. Data are presented as mean±SD. *P < .05, **P < .01

It has been clarified that AFAP-AS1 was dysregulated in different types of cancer. To elucidate the function of AFAP-AS1 in thyroid tumorigenesis, we first analyzed the expression of AFAP-AS1 in thyroid cancer cells and immortalized thyroid cells. As shown in Figure 1(a), the level of AFAP-AS1 in thyroid cancer cells 8305 C, 8505 C and C643 were over-expressed than immortalized and non-tumorigenic thyroid cell Nthy-ori-3-1. Next the FISH assays were used to detect the location of AFAP-AS1. Our data indicated that AFAP-AS1 was located mainly in cytoplasm but not nuclear in 8305 C and 8505 C cells (Figure 1(b)). Therefore, we demonstrated that AFAP-AS1 was dysregulated in ATC cells and located in cytoplasm.

### 3.2. Knockdown of AFAP-AS1 suppressed the progression thyroid cancer cells

**Figure 2.**
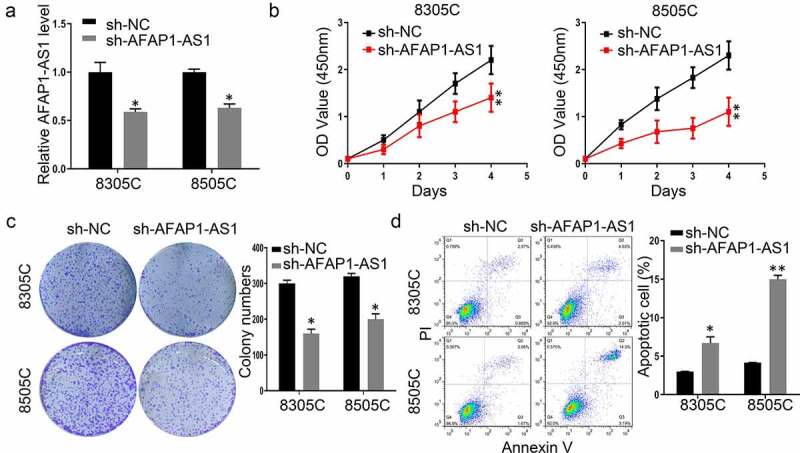
Knockdown of AFAP-AS1 suppressed the progression thyroid cancer cells. (a), The RNA expression of AFAP-AS1 after lentivirus sh-negative control (sh-NC) or sh-AFAP-AS1 transfected into 8305 C and 8505 C cell. (b), CCK8 experiments examined the proliferation rate of 8305 C and 8505 C after sh-NC or sh-AFAP-AS1 transfection. (c), The colony numbers were counted after sh-NC or sh-AFAP-AS1 transfected into 8305 C and 8505 C cell. (d), The percentage of apoptotic cells were evaluated by flow cytometry after sh-NC or sh-AFAP-AS1 transfected into 8305 C and 8505 C cell. Data are presented as mean±SD. *P < .05, **P < .01

To explore the function of AFAP-AS1 in thyroid cancer, shRNAs were used to deplete the expression of AFAP-AS1 in 8305 C and 8505 C cells. As shown in Figure 2(a), the expression of AFAP-AS1 decreased after sh-AFAP-AS1 transfection. Then we performed CCK8 assays to investigate the effect of AFAP-AS1 on cell growth. The results demonstrated that knockdown of AFAP-AS1 inhibited the proliferation of 8305 C and 8505 C (Figure 2(b)). The inhibitory effect of AFAP-AS1 on thyroid cancer progression was further confirmed by colony formation assays. It was shown that the numbers of colony in sh-AFAP-AS1 transfected cells were fewer than those formed in sh-NC cells (Figure 2(c)). In addition, the apoptotic rate increased after transfected with sh-AFAP-AS1 in 8305 C and 8505 C cells (Figure 2(d)). Thus, our results proved that knockdown of AFAP-AS1 inhibited proliferation and enhanced apoptosis in thyroid cancer cells.

### 3.3. AFAP-AS1 regulate the expression of ETS1 through sponging miR-155-5p

**Figure 3.**
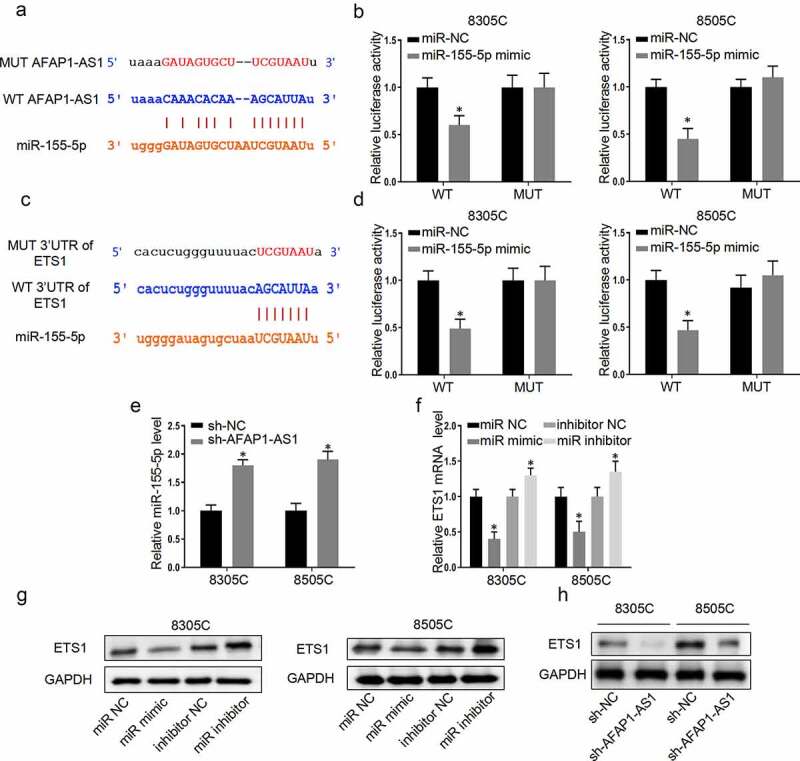
AFAP-AS1 regulate the expression of ETS1 through sponging miR-155-5p. (a), The bioinformatic analysis predicted the binding site between AFAP-AS1 and miR-155-5p. The mutated sites of AFAP-AS1 were shown. (b), Wild type of AFAP-AS1 plasmid (WT) and mutant type of AFAP-AS1 plasmid (MUT) were transfected into 8305 C and 8505 C cells. Then luciferase activity was assessed in 8305 C cells after miR-NC or miR-155-5p transfection. (c), The bioinformatic analysis predicted the binding site between 3ʹ-UTR-ETS1 and miR-155-5p. The mutated sites of 3ʹUTR-ETS1 were shown. (d), Wild type of 3ʹ-UTR-ETS1 (WT) plasmid or mutant type of 3ʹ-UTR-ETS1 (MUT) plasmid were transfected into 8305 C and 8505 C cells. Then luciferase activity was examined after miR-NC or miR-155-5p transfection. (e), The expression level of miR-155-5p was assessed in 8305 C and 8505 C cells after sh-NC or sh-AFAP-AS1 transfection. U6 as an internal control. (f), The miR-negative control (miR-NC), miR-155-5p mimic (miR-mimic), inhibitor-negative control (inhibitor-NC) or miR-155-5p inhibitor (miR-inhibitor) were transfected into 8305 C and 8505 C cells. Then the mRNA levels of ETS1 were examined. GAPDH as an internal control. (g), Western blot was used to detect the expression of ETS1 when transfected with miR-NC, miR-155-5p mimic, inhibitor-NC or miR-155-5p inhibitor. GAPDH used as internal control. (h), The protein levels of ETS1 in 8305 C and 8505 C cells after sh-NC or sh-AFAP-AS1 transfection. GAPDH used as internal control. Data are presented as mean±SD. *P < .05

As is known to us that lncRNA which located in cytoplasm exerted its function through sponging microRNA, bioinformatic analysis (http://starbase.sysu.edu.cn/) was used to predict that AFAP-AS1 could bind to miR-155-5p. Then the wild type of AFAP-AS1 plasmid (WT-AFAP-AS1) and mutant type of AFAP-AS1 plasmid (MUT-AFAP-AS1) were constructed to perform luciferase reporter assays (Figure 3(a)). The results demonstrated miR-155-5p mimic only impaired the luciferase activity of the WT-AFAP-AS1, but not MUT-AFAP-AS1 in 8305 C cells and 8505 C cells. However, miR-NC had no effect on luciferase activity both in WT-AFAP-AS1 and MUT-AFAP-AS1 in 8305 C cells and 8505 C cells (Figure 3(b)). Meanwhile, the binding effects of miR-155-5p on ETS1 promotor was predicted through bioinformatic analysis (http://starbase.sysu.edu.cn/). Then wild type of 3ʹ-UTR-ETS1 plasmid (WT-3ʹUTR-ETS1) and mutant type of 3ʹ-UTR-ETS1 plasmid (MUT-3ʹUTR-ETS1) were constructed to perform luciferase reporter assays (Figure 3(c)). The luciferase reporter assays were used to confirm that miR-155-5p decreased the luciferase activities of WT-3ʹUTR-ETS1while there was no significant influence on 8305 C cells and 8505 C cells with MUT-3ʹUTR-ETS1. Next, we further explored the relationship between AFAP-AS1 and miR-155-5p. Depletion of AFAP-AS1 markedly caused an increase in the expressions of miR-155-5p in 8305 C and 8505 C cells (Figure 3(e)). This data supported that AFAP-AS1 negatively regulated the expressions of miR-155-5p. To investigate the binding effect between miR-155-5p and ETS1, we assessed the RNA and protein expression of ETS1 after transfected with miR-155-5p mimic or a miR-NC or miR-inhibitor-NC (100 pmol) or miR-155-5p-inhibitor. The results demonstrated that over-expression of miR-155-5p decreased the expression of protein and RNA of ETS1, while inhibition of miR-155-5p had an inverse effect (figure 3(f and figure 3g)). In addition, the results showed that knockdown of AFAP-AS1 reduced the protein expression of ETS1 (Figure 3(h)). Thus, we proved that lncAFAP-AS1 regulate the expression of ETS1 through sponging miR-155-5p.

### 3.4. AFAP-AS1 exerted its oncogene function through miR-155-5p/ETS1/ERK pathway

**Figure 4.**
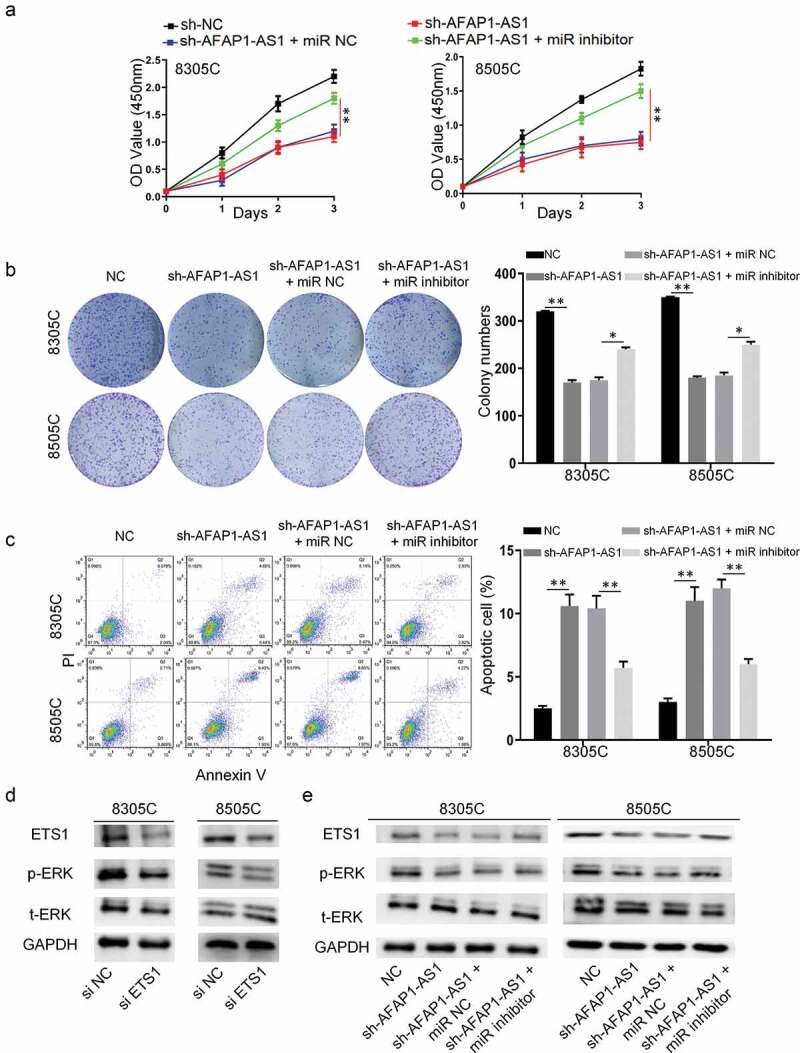
AFAP-AS1 exerted its oncogene function through miR-155-5p/ETS1/ERK pathway. 8305 C and 8505 C cells were transfected with sh-NC or sh-AFAP-AS1 or sh-AFAP-AS1 and miR-NC or sh-AFAP-AS1 and miR-155-5p inhibitor. (a),The the cell viability was detected by CCK-8 assays. (b), The colony numbers were counted. (c), The apoptosis of cells was evaluated by flow cytometry. (d), The western blot showed the protein levels of ETS1, p-ERK, t-ERK and GAPDH in 8305 C and 8505 C cell after siRNA negative control (si-NC) or siRNA-ETS1 (si-ETS1) transfection. (e), Western blot showed the protein levels of ETS1, p-ERK, t-ERK and GAPDH in 8305 C and 8505 C cell transfected with sh-NC, sh-AFAP-AS1, sh-AFAP-AS1 and miR-NC or sh-AFAP-AS1 and miR-155-5p inhibitor. Data are presented as mean±SD. *P < .05, **P < .01

To confirm AFAP-AS1 play an oncogene role through miR-155-5p in thyroid cancer, we used rescue experiments that transfected miR-155-5p mimic or miR-NC after AFAP-AS1 depletion. As shown in Figure 4(a), miR-155-5p inhibitor, but not miR-NC, reversed inhibitory effect of AFAP-AS1 depletion on cell viability in 8305 C and 8505 C cells. Meanwhile, colony formation experiments also proved this restoration mentioned above (Figure 4(b)). Moreover, there was also an attenuation of apoptosis when miR-155-5p inhibitor and sh-AFAP-AS1 transfected into 8305 C and 8505 C cells together(Figure 4(c)). Previous researches showed that the ERK signal pathway played an important role in the pathological behavior of anaplastic thyroid cancer. Next, we examined the protein level of pERK when cells were transfected with si-ETS1. The results showed that knockdown of ETS1 suppressed ERK phosphorylation, but not total ERK expression (Figure 4(d)). In addition, ETS1/ERK pathway in 8305 C and 8505 C cells were evaluated after knockdown of AFAP-AS1 to investigate the underlying mechanism. As expected, the western blot results showed that ETS1 and pERK declined when cells were transfected with sh-AFAP-AS1. Then the rescue experiments also performed to prove AFAP-AS1 regulate ETS1/pERK through miR-155-5p. The results showed that miR-155-5p inhibitor reversed the decreased protein level of ETS1 and pERK when AFAP-AS1 depletion in 8305 C and 8505 C cells (Figure 4(e)). Thus, we observed that AFAP-AS1 exerted its oncogene function through miR-155-5p/ETS1/ERK pathway.

### 3.5. *Knockdown of AFAP-AS1 inhibited tumorigenesis of thyroid* in vivo

**Figure5.**
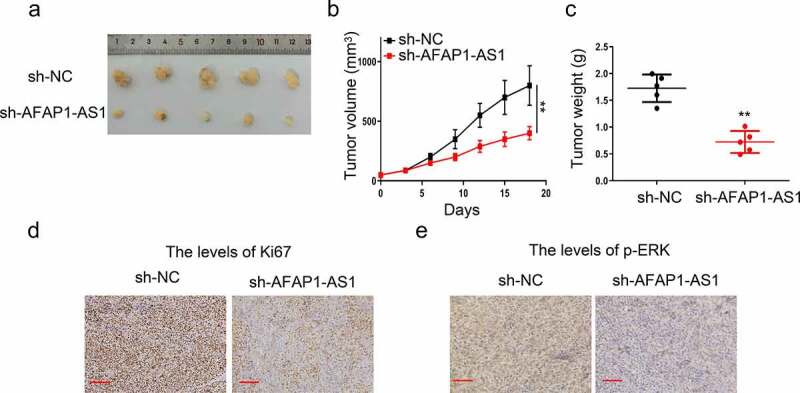
Knockdown of AFAP-AS1 inhibited tumorigenesis of thyroid *in vivo*. 8305 C cells were transfected with sh-NC and sh-AFAP-AS1 and then were injected into nude mice. (a), Representative images of mice bearing tumors. (b), Tumor volumes were measured every three days, and growth curves are shown. (c), Tumor weight were measured. (d), Representative images for Ki67 immunostaining of tumor tissues were shown. Scale bar = 200um. (e), Representative images for p-ERK immunostaining of tumor tissues were shown. Scale bar = 100um. Data are presented as mean±SD. **P < .01

Finally, we evaluated oncogene effect of AFAP-AS1 in nude mice by 8305 C tumor xenograft. As shown in Figure 5(a–Figure 5c), knockdown of AFAP-AS1 significantly reduce the tumor volume and weight in contrast to the control group. Then we used immunohistochemical methods to examine the expression of Ki67 and pERK in tissues. The results demonstrated that the percentage of Ki67-positive cells strinkingly after AFAP-AS1 depletion (Figure 5(d)). Meanwhile, we also observed there was an attenuation of pERK expression after AFAP-AS1 depletion (Figure 5(e)). Therefore, we proved that depletion of AFAP-AS1 inhibited the progression of anaplastic thyroid cancer *in vivo*.

## 4. Discussion

As anaplastic thyroid cancer is the most malignant type of thyroid cancer, there was compelling need to figure out a biomarker to predict its process. Previous research indicated that AFAP-AS1 facilitated to predict the diagnosis and prognosis of patients with cancer as a potential biomarker [26,27]. However, the function of AFAP-AS1 in ATC remained unknown. In this study, we explored the function and mechanism of AFAP-AS1 in ATC. It has been reported that AFAP-AS1 participated in cell proliferation, dedifferentiation and metastasis of several types of cancer including gastric cancer, nasopharyngeal carcinoma, non-small cell lung cancer [12,13,20]. Consistent with previous study showed that AFAP-AS1 exerted oncogene role, our study firstly showed that AFAP-AS1 over-expressed in ATC cells compared to normal cells. Meanwhile, *vitro* and *in vivo* experiments were performed to prove its oncogene role in anaplastic thyroid cancer. When AFAP-AS1 was knocked down, the proliferation of ATC cell lines and xenograft to nude mice were inhibited significantly.

As is known to us, lncRNAs exert its regulatory effect mainly by absorbing the target miRNA so this miRNA cannot bind to the corresponding gene. Therefore, we speculated that AFAP-AS1 participated in ATC progression through this mechanism. Bioinformatical analysis and biological experiments were used to hypothesized that AFAP-AS1 bound to miR-155-5p. The luciferase activities proved that binding effect between AFAP-AS1 and miR-155-5p. Meanwhile, knockdown of AFAP-AS1 significantly caused an increase in miR-155-5p expressions in 8305 C and 8505 C cells. We confirmed that miR-155-5p negatively regulated by AFAP-AS1. Previous studies showed that controversial results about the function of miR-155-5p in thyroid cancer progression. Rezaei et al. indicated that there was no significant difference of miR-155-5p expression between thyroid cancer and normal tissues while Wylie et al. demonstrated that the expression of miR-155-5p in ATC was higher than other carcinoma [28,29]. Thus, to prove whether AFAP-AS1 regulate ATC proliferation through miR-155-5p, we performed the rescue experiment that added miR-155-5p inhibitor when AFAP-AS1 was knocked down in ATC. The investigation presented that miR-155-5p inhibitor reversed proliferation inhibition after AFAP-AS1 depletion. These evidences indicated that ATC with high AFAP-AS1 expression accelerated ATC process because it absorbed miR-155-5p as sponge to prevent miR-155-5p post-transcriptional modification of target gene.

Then bioinformatics technology was performed to screen the downstream target genes of miR-155-5p and we selected ETS1 which was controlled by several miRNAs. It has been reported that ETS1-dependent oncogenic transformation was mediated by miR17-92 clusters [30]. Meanwhile, current studies showed that miR-9 inhibited gastric cancer and miR-512-5p impaired non-small cell lung cancer proliferation through targeting ETS1 [31]. Similar to the research mentioned above, we demonstrated that miR-155-5p could regulated ETS1 through post-transfection. Over-expressions of miR-155-5p decreased the expression of protein levels of ETS1 while inhibition of miR-155-5p had an inverse effect.

Previous researches showed that as a transcription factor, the downstream of ETS1 were several classic tumor-related genes, such as RAS, MET, ERK and etc [21]. It has been reported that the ERK signal pathway played a crucial part in the pathological behavior of anaplastic thyroid cancer and directly influence the prognosis of anaplastic thyroid cancer. Unlike the previous study showed that ETS1 impact the protein expression of ERK [23], we found that ETS1 influence ERK phosphorylation but not its expression.

From the above data, we drew a conclusion: AFAP-AS1 exerted its oncogene function through adsorbing miR-155-5p as sponge to regulate ETS1 which correlated to ERK pathway. However, there are still potential mechanisms that need to be discussed as followings. Several reports showed other three mechanisms that lncRNA participated in physiological and pathological process except adsorbing microRNA as sponge. Firstly, certain specific lncRNA recruited chromatin remodeling and modification complexes to target sites, altering methylation status of DNA, RNA and histone in order to control the expression of related genes. In this way, it may result in the occurrence of certain diseases such as cancer because of loss of expression [32]. Secondly, lncRNA acted as ligands and combined with some transcription factors to form complexes that regulate gene expression [33,34]. Thirdly, lncRNA also directly participates in the post-transcriptional regulation of mRNA, including variable splicing, RNA editing and protein translation. Antisense lncRNA is mainly involved in post-transcriptional regulation of mRNA, which combines with the complementary region of mRNA and affects the recruitment of spliceosome at certain splicing sites and controls the process of mRNA splicing [35]. Therefore, we speculated that whether AFAP-AS1 exerted its oncogene role in ATC through these three mechanisms talked above needed to be identified in the further experiments.

The novelty of our research is that we initially discovered that depletion of AFAP-AS1 could suppress ATC progression and explored its molecular mechanism. Compared to previous study [13,36], we demonstrated AFAP-AS1 negatively regulate miR-155-5p but not other miRNAs. We subsequently proved that AFAP-AS1 exerted its oncogene role through miR-155-5p/ETS1/ERK pathway.

## 5. Conclusion

In this study, we observed that AFAP-AS1 over-expressed in anaplastic thyroid cancer cells compared to immortalized thyroid cells. Depletion of AFAP-AS1 inhibited proliferation and enhanced apoptosis in anaplastic thyroid cancer. Subsequently, a series of bioinformatic technologies and molecular experiments indicated that miR-155-5p as the target gene of AFAP-AS1 influenced tumor progress by regulating the classic tumor- related transcription factor ETS1. Next, we proved that ETS1 took an important part in the progression of anaplastic thyroid cancer by controlling Erk phosphorylation. Compared to previous study, we initially demonstrated that depletion of AFAP-AS1 inhibited progression of anaplastic thyroid cancer through miR-155-5p/ETS1/Erk pathway.

## Highlights

1. Knockdown of AFAP-AS1 suppressed the progression of anaplastic thyroid cancer cells.
2. LncAFAP-AS1 regulated the expression of ETS1 through sponging miR-155-5p.
3. LncAFAP-AS1 exerted its oncogene function through miR-155-5p/ETS1/ERK pathway.
4. Knockdown of AFAP-AS1 inhibited growth of xenograft derived from anaplastic thyroid cancer *in vivo.*

## References

[1] Siegel RL, Miller KD, Jemal A. Cancer statistics, 2018. CA Cancer J Clin. 2018;68(1):7–30. 29313949 10.3322/caac.21442

[2] Kitahara CM, Sosa JA. The changing incidence of thyroid cancer. Nat Rev Endocrinol. 2016;12(11):646–653. 27418023 10.1038/nrendo.2016.110 PMC10311569

[3] Viola D, Valerio L, Molinaro E, *et al*. Treatment of advanced thyroid cancer with targeted therapies: ten years of experience. Endocr Relat Cancer. 2016;23(4):R185–205. 27207700 10.1530/ERC-15-0555

[4] Schneider DF, Chen H. New developments in the diagnosis and treatment of thyroid cancer. CA Cancer J Clin. 2013;63(6):374–394. 23797834 10.3322/caac.21195 PMC3800231

[5] Gentile D, Orlandi P, Banchi M, et al. Preclinical and clinical combination therapies in the treatment of anaplastic thyroid cancer. Med Oncol. 2020;37(3):19. 32108281 10.1007/s12032-020-1345-2

[6] Lowe NM, Loughran S, Slevin NJ, et al. Anaplastic thyroid cancer: the addition of systemic chemotherapy to radiotherapy led to an observed improvement in survival–a single centre experience and review of the literature. Sci World J. 2014;2014:674583. 10.1155/2014/674583 PMC3947878 25184150

[7] Fallahi P, Ruffilli I, Elia G, et al. Novel treatment options for anaplastic thyroid cancer. Expert Rev Endocrino. 2017;12(4):279–288. 10.1080/17446651.2017.1340155 30058884

[8] Al-Jundi M, Thakur S, Gubbi S, et al. Novel targeted therapies for metastatic thyroid cancer-A comprehensive review. Cancers (Basel). 2020;12(8). 10.3390/cancers12082104 PMC7463725 32751138

[9] Mi C, Zhang CC, Zhang Q, et al. Increased expression of lncRNA HULC in human epithelial ovarian cancer and its biological functions. Eur J Gynaecol Oncol. 2018;39(6):992–996.

[10] Liu X, Fu Q, Li S, et al. LncRNA FOXD2-AS1 functions as a competing endogenous RNA to regulate TERT expression by sponging miR-7-5p in thyroid cancer. Front Endocrinol (Lausanne). 2019;10:207. 31024447 10.3389/fendo.2019.00207 PMC6463795

[11] Zhao H, Zhang K, Wang T, et al. Long non-coding RNA AFAP1-antisense RNA 1 promotes the proliferation, migration and invasion of gastric cancer cells and is associated with poor patient survival. Oncol Lett. 2018;15(6):8620–8626. 29805596 10.3892/ol.2018.8389 PMC5950603

[12] Bo H, Gong ZJ, Zhang WL, *et al*. Upregulated long non-coding RNA AFAP1-AS1 expression is associated with progression and poor prognosis of nasopharyngeal carcinoma. Oncotarget. 2015;6(24):20404–20418. 26246469 10.18632/oncotarget.4057 PMC4653014

[13] Huang N, Guo W, Ren K, et al. LncRNA AFAP1-AS1 supresses miR-139-5p and promotes cell proliferation and chemotherapy resistance of non-small cell lung cancer by competitively upregulating RRM2. Front Oncol. 2019;9. DOI:10.3389/fonc.2019.01103 PMC6817562 31696057

[14] Wang YM, Mo YZ, Yang X, *et al*. Long non-coding RNA AFAP1-AS1 is a novel biomarker in various cancers: a systematic review and meta-analysis based on the literature and GEO datasets. Oncotarget. 2017;8(60):102346–102360. 29254250 10.18632/oncotarget.21830 PMC5731960

[15] Tan K, Ge Y, Tian J, et al. miRNA-9 inhibits apoptosis and promotes proliferation in angiotensin II-induced human umbilical vein endothelial cells by targeting MDGA2. Rev Cardiovasc Med. 2019;20(2):101–108. 31345003 10.31083/j.rcm.2019.02.514

[16] Hu Y, Liu Q, Zhang M, et al. MicroRNA-362-3p attenuates motor deficit following spinal cord injury via targeting paired box gene 2. J Integr Neurosci. 2019;18(1):57–64. 31091849 10.31083/j.jin.2019.01.12

[17] Sun Y, Chen G, He J, *et al*. Clinical significance and potential molecular mechanism of miRNA-222-3p in metastatic prostate cancer. Bioengineered. 2021;12(1):325–340. 33356818 10.1080/21655979.2020.1867405 PMC8806336

[18] Li YP, Du XR, Zhang R, et al. Interleukin-18 promotes the antitumor ability of natural killer cells in colorectal cancer via the miR-574-3p/TGF-beta1 axis. Bioengineered. 2021;12(1):763–778. 33660570 10.1080/21655979.2021.1880717 PMC8806203

[19] Li N, Cui T, Guo WL, et al. MiR-155-5p accelerates the metastasis of cervical cancer cell via targeting TP53INP1. Oncol Targets Ther. 2019;12::3181–3196. 10.2147/OTT.S193097 PMC6500876 31118671

[20] Ma HW, Xi DY, Ma JZ, et al. Long noncoding RNA AFAP1-AS1 promotes cell proliferation and metastasis via the miR-155-5p/FGF7 axis and predicts poor prognosis in gastric cancer. Dis Markers. 2020;2020:8140989. 32051698 10.1155/2020/8140989 PMC6995499

[21] Dittmer J. The role of the transcription factor Ets1 in carcinoma. Semin Cancer Biol. 2015;35::20–38. 10.1016/j.semcancer.2015.09.010 26392377

[22] Zhai W, Ma JJ, Zhu RJ, *et al*. MiR-532-5p suppresses renal cancer cell proliferation by disrupting the ETS1-mediated positive feedback loop with the KRAS-NAP1L1/P-ERK axis. Br J Cancer. 2018;119(5):591–604. 30082686 10.1038/s41416-018-0196-5 PMC6162242

[23] Peyret V, Nazar M, Martin M, *et al*. Functional toll-like receptor 4 overexpression in papillary thyroid cancer by MAPK/ERK-induced ETS1 transcriptional activity. Mol Cancer Res. 2018;16(5):833–845. 29523762 10.1158/1541-7786.MCR-17-0433

[24] Choi C, Tran NTT, Ngu TV, *et al*. Promotion of tumor progression and cancer stemness by MUC15 in thyroid cancer via the GPCR/ERK and integrin-FAK signaling pathways. Oncogenesis. 2018;7. DOI:10.1038/s41389-018-0094-y. PMC6232104 30420637

[25] Boussemart L, Malka-Mahieu H, Girault I, et al. eIF4F is a nexus of resistance to anti-BRAF and anti-MEK cancer therapies. Nature. 2014;513(7516):105-+. 10.1038/nature13572 25079330

[26] Ji DL, Zhong XY, Jiang XM, et al. The role of long non-coding RNA AFAP1-AS1 in human malignant tumors. Pathol Res Pract. 2018;214(10):1524–1531. 30173945 10.1016/j.prp.2018.08.014

[27] Wang Y, Zhou L, Lu J, et al. Research progress on long non-coding RNAs and their roles as potential biomarkers for diagnosis and prognosis in pancreatic cancer. Cancer Cell Int. 2020;20:457. 32973402 10.1186/s12935-020-01550-y PMC7493950

[28] Rezaei M, Khamaneh AM, Zarghami N, et al. Evaluating pre- and post-operation plasma miRNAs of papillary thyroid carcinoma (PTC) patients in comparison to benign nodules. BMC Cancer. 2019;19(1):690. 31307429 10.1186/s12885-019-5849-0 PMC6631438

[29] Wylie D, Beaudenon-Huibregtse S, Haynes BC, et al. Molecular classification of thyroid lesions by combined testing for miRNA gene expression and somatic gene alterations. J Pathol Clin Res. 2016;2(2):93–103. 27499919 10.1002/cjp2.38 PMC4907059

[30] Kabbout M, Dakhlallah D, Sharma S, et al. MicroRNA 17-92 cluster mediates ETS1 and ETS2-dependent RAS-oncogenic transformation. PloS One. 2014;9(6):e100693. 24968297 10.1371/journal.pone.0100693 PMC4072627

[31] Cao B, Tan S, Tang H, et al. miR‑512‑5p suppresses proliferation, migration and invasion, and induces apoptosis in non‑small cell lung cancer cells by targeting ETS1. Mol Med Rep. 2019. DOI:10.3892/mmr.2019.10022 PMC6471623 30896817

[32] Wei JW, Huang K, Yang C, et al. Non-coding RNAs as regulators in epigenetics. Oncol Rep. 2017;37(1):3–9. 27841002 10.3892/or.2016.5236

[33] Long YC, Wang XY, Youmans DT, et al. How do lncRNAs regulate transcription? Sci Adv. 2017;3(9). DOI:10.1126/sciadv.aao2110 PMC5617379 28959731

[34] Dykes IM, Emanueli C. Transcriptional and post-transcriptional gene regulation by long non-coding RNA. Genom Proteom Bioinf. 2017;15(3):177–186. 10.1016/j.gpb.2016.12.005 PMC5487525 28529100

[35] Wilusz JE. Long noncoding RNAs: re-writing dogmas of RNA processing and stability. Bba-Gene Regul Mech. 2016;1859(1):128–138. 10.1016/j.bbagrm.2015.06.003 PMC4676738 26073320

[36] Zhong YW, Wang YD, Dang HM, et al. LncRNA AFAP1-AS1 contributes to the progression of endometrial carcinoma by regulating miR-545-3p/VEGFA pathway. Mol Cell Probe. 2020; 53. 10.1016/j.mcp.2020.101606 32504788

